# VIRTUES: A Virtual Reality Multimodal Sensing Platform for Quantifying and Supporting Cross-Neurotype Collaboration

**DOI:** 10.3390/s26092906

**Published:** 2026-05-06

**Authors:** Ashwaq Zaini Amat, Mahrukh Tauseef, Deeksha Adiani, Amy S. Weitlauf, Nilanjan Sarkar

**Affiliations:** 1 Department of Electrical and Computer Engineering, Vanderbilt University, Nashville, TN 37240, USA; 2 Department of Computer Science, Vanderbilt University, Nashville, TN 37240, USA; deeksha.m.adiani@vanderbilt.edu; 3 Treatment and Research Institute for Autism Spectrum Disorders (TRIAD), Vanderbilt University Medical Center, Nashville, TN 37212, USA; 4 Department of Mechanical Engineering, Vanderbilt University, Nashville, TN 37240, USA

**Keywords:** collaborative virtual environment, multimodal sensing, collaborative learning tools, real-time feedback mechanism, cross-neurotype collaboration

## Abstract

Effective workplace collaboration is essential for productivity and creativity, yet achieving the necessary mutual understanding can be challenging, particularly involving individuals from different neurotypes. This work evaluates VIRTUES, a Virtual Reality (VR) platform designed to foster mutual understanding and collaborative behaviors between autistic and neurotypical individuals. VIRTUES integrates multimodal sensing (eye tracking, interaction logs, and transcribed speech) to objectively quantify five defined dimensions of collaboration while providing real-time, context-aware support through an embedded rule-based feedback mechanism. A user study involving 12 autistic–neurotypical pairs demonstrates that VIRTUES can assess and support collaborative efforts across different neurotypes. Through synchronized sensing data, we identified that Information Pooling serves as a critical driving factor for successful collaborative performance. These preliminary findings suggest that VIRTUES provides a foundation for exploring inclusive teamwork and may inform the design of future interventions to support neurodiverse social-technical skill acquisition.

## 1. Introduction

Workplaces are becoming increasingly inclusive, emphasizing the value of collaboration among individuals with diverse cognitive profiles, including cross-neurotype collaboration [1]. Cross-neurotype collaborative interactions, such as team meetings and teamwork involving autistic and neurotypical colleagues, can enhance productivity, innovation and workplace performance due to the diverse perspectives and inclusivity [1,2,3]. Despite its benefits, cross-neurotype collaboration presents unique challenges [4,5], particularly due to differences in communication and collaboration styles as highlighted by the Double Empathy Problem (DEP) [6]. DEP posits that communication and teamwork challenges between autistic and neurotypical individuals arise from mutual misunderstandings, and a mismatch in social expectations, rather than deficits of one group. Therefore, there is a need for both neurotypical and neurodiverse individuals to develop strategies to adapt and improve their collaborative interactions [7,8,9].

Simulation-based training (SBT) has been shown to be effective in improving collaborative skills [9,10,11]. SBT enables individuals in a team to engage in a shared experience that involves social, cognitive, and behavioral exchange to achieve a common objective. Most SBT programs are done in-person and observed by trainers that provide feedback on their teamwork performance either in real-time or in a post-training session. Although SBTs have been proven effective in developing teamwork skills, they are often resource-intensive and may not fully capture the complexities of cross-neurotype collaborative interactions [12]. On the other hand, existing training programs designed with neurodiverse individuals in mind often focus on ‘correction’ of neurodiverse behavior, often placing disproportionate pressure on neurodivergent individuals [6,7]. It has been reported that neurotypical partners play a crucial role as active participants in successful collaborative interactions [13]. There is a need for a training environment that is inclusive for neurodiverse and neurotypical individuals to equally train and develop their collaborative skills.

Collaborative virtual environments (CVEs) can be leveraged to allow multiple individuals to share an interactive and adaptive virtual space that can simulate any training environment. By simulating realistic environments in a controlled, repeatable, and data-rich virtual space, neurodiverse and neurotypical individuals can engage in adaptive, task-based scenarios where communication and coordination can be practiced, fostering more effective communication and mutual understanding [8,14]. CVE-based SBT would enable inclusive training experiences through equitable collaboration to enhance cross-neurotype collaboration, ensuring that both autistic and neurotypical individuals can develop the skills needed for successful collaboration. Complex social skills such as teamwork can be challenging to assess [11]. Existing methods of assessment still rely heavily on human observations [15,16]. In contrast, this work proposes a multimodal sensing framework that systematically maps multimodal sensing data to theoretically grounded dimensions of collaboration, enabling quantification of collaborative interaction. These multimodal sensing data can also be used to frame a feedback mechanism that can scaffold collaborative interactions [17,18].

Recent studies on CVE-based interactions show promising outcomes. Research focusing on social communication skills has demonstrated improvements in emotion recognition and conversational abilities among autistic users who engage in structured social scenarios within CVEs [16,19]. Comparative work further indicates that participants in CVEs exhibit more frequent verbal exchanges than those in face-to-face settings when performing the same tasks [20]. More recently, integrated interventions combining social communication and motor skills training have led to enhanced performance across both domains for autistic children [21]. While existing CVE-based SBTs have explored collaborative interactions across multiple domains, there are not many that focus on cross-neurotype interaction.

Recognizing the clear need for a cross-neurotype collaborative skills training platform, we present a novel collaborative VIRtual environment for Teamwork and Executive functioning skills Simulator (VIRTUES) as a virtual training tool in cross-neurotype collaborative interactions. VIRTUES builds on our prior works evaluating the feasibility and acceptability of a series of collaborative tasks suitable for dyadic collaborative interactions [22,23]. In this present work, we substantially extend the state of the art in both design and evaluation of our system guided by the following three hypotheses:

**H1.**  *VIRTUES and its feedback mechanism will positively drive and support collaborative interactions within cross-neurotype dyads during the task.*

**H2.**  *The multimodal sensing features can be objectively mapped to the five dimensions of collaboration, providing a richer dyadic profile than single-modality metrics.*

**H3.**  *VIRTUES assessment tasks can successfully measure variations and changes in collaboration dimensions for all participants.*

The primary contributions of this work are as follows: (1) We define and operationalize five core dimensions of collaboration and establish a unique mapping framework that translates synchronized multimodal sensor data into these objective performance metrics; (2) introduced a rule-based feedback system that utilizes real-time sensor data to provide active, situated support designed to facilitate mutual understanding in neurodiverse dyads; (3) demonstrate that the assessment tasks effectively capture collaborative shifts for both autistic and neurotypical individuals, establishing the framework’s utility as a universal and unbiased tool for inclusive workforce evaluation; and (4) a user study involving 12 autistic–neurotypical pairs that evaluates the feasibility of VIRTUES and identifying key driving factors of successful collaboration.

The remainder of the paper is organized as follows: Section 2 presents the VIRTUES system design and describes our experimental setup and hypotheses. Section 3 details our data analysis method. Section 4 presents the results, followed by Section 5, which includes the discussion of our analysis. Section 6 summarizes our findings, addresses the limitations and outlines the future directions for this research.

## 2. System Design

### 2.1. VIRTUES System Architecture

**Figure 1:**
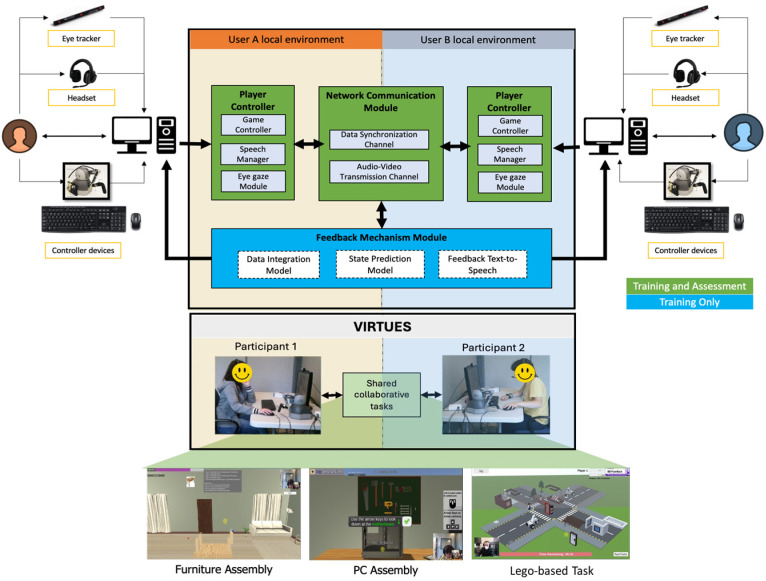
System architecture for VIRTUES collaborative training and assessment tasks with setup where two participants in separate rooms perform virtual collaborative tasks.

The architecture of VIRTUES together with the system setup is shown in Figure 1.

*The hardware:* VIRTUES was designed to operate on two standard desktop computers, with the same specifications, equipped with Windows 10 Education, with an Intel Xeon E5-1650 CPU @3.20 GHz, and 16 GB of RAM, with a 28-inch LCD with 1920 × 1080 resolution running at 60 Hz. The interaction modalities that were used to communicate and navigate the virtual environment consisted of a headset (speaker with microphone) and webcam for audiovisual communication between dyads, an eye tracker to detect participants’ gaze in the virtual space, and task-dependent controllers to manipulate virtual objects and navigate the virtual space. Through the sensors and peripheral devices, 15 parameters were collected (see Table 1) as quantitative multimodal sensing data.

*The software:* The collaborative tasks were developed using Unity 2021.3.8f1 [24], a multi-platform game development software. A Player Controller component enables participants to interact with the system through the input device while continuously tracking task progression and timing. This component consists of three sub-modules: (1) a Game Controller that manages user interactions with virtual objects while recording task-related actions, (2) a Speech Manager that captures and transcribes participants’ spoken communication in real time using Microsoft Azure’s Speech-to-Text service [25], allowing extraction of utterance-level features such as word count and duration, and (3) an Eye Gaze Module that utilizes a Tobii EyeX 2.0.4 [26] tracker to monitor gaze behavior and detect fixations on predefined regions of interest within the virtual environment. All multimodal data—including controller interaction, speech, and gaze—are time-synchronized and transmitted to the Data Synchronization Channel for subsequent analysis. The Network Communication Module enables two participants to connect and interact within a shared virtual environment by managing real-time synchronization, audio, and video communication. Object synchronization is handled using the Mirror Networking [27] over TCP to ensure reliable and ordered data transmission, while audio and video streaming are supported via WebRTC API [28] using UDP to prioritize low latency. The latency does not significantly affect task interaction since our tasks do not require instantaneous updates.

A feasibility study was conducted to evaluate the collaborative tasks used in this work [22,23]. Section 2.3 summarizes the design of each task based on a set of defined dimensions of collaboration discussed in Section 2.2.

### 2.2. Dimensions of Collaboration

There are currently no standardized methods to measure collaboration skills in group interactions. Meier et al. [29] identified nine collaboration dimensions in a video conferencing system focusing on problem-solving, joint information processing, motivation, and coordination between two groups of students. In our previous analysis [22], we found that the collaboration dimensions defined by Meier et al. [29] focused primarily on verbal communication, overlooking useful non-verbal components such as physical coordination and spatial dynamics. Given the importance of these elements in collaborative interactions and the nature of our collaborative interaction simulator, VIRTUES, we redefined the set of collaboration dimensions (Table 2) which reflect the collaborative interactions within our training environment better.

### 2.3. Collaborative Tasks

We designed the collaborative tasks in VIRTUES using a participatory approach, actively involving key stakeholders, including a certified behavioral analyst, human resource representatives from two companies, career counselors from two vocational rehabilitation centers, and three autistic individuals. Their insights and review of relevant literature informed the development of two cross-neurotype training tasks [22]: PC assembly [30] and furniture assembly [31], and one LEGO assessment task [32,33,34].

#### 2.3.1. Training Tasks

The detailed design of the PC Assembly and furniture assembly is discussed in [22]. However, in this work, we summarize the task descriptions together with incorporated dimensions of collaboration in Table 3.

#### 2.3.2. Pre- and Post-Assessment Tasks

We developed pre- and post-assessment tasks designed to measure the effectiveness of the training activities. These tasks incorporated the five dimensions of collaboration from Table 2 and used the same system architecture (Figure 1), excluding the Feedback Mechanism Module. The pre-assessment task established a baseline for participants’ collaborative performance, while the post-assessment task measured any changes after training. Two variations in the assessment activity were developed to prevent habituation effects.

**Figure 2:**
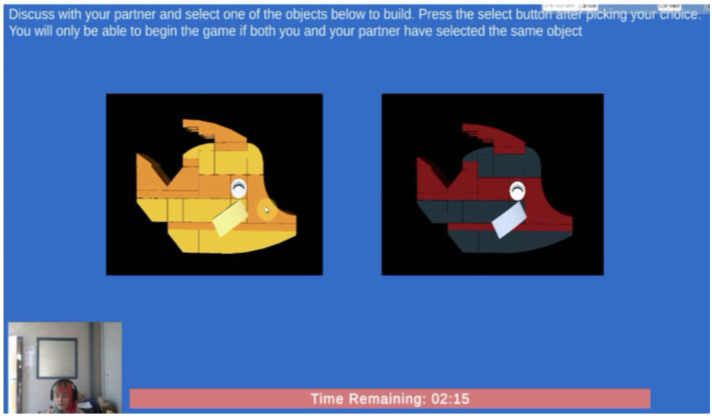
The assessment task showing two design options they need to choose from.

**Figure 3:**
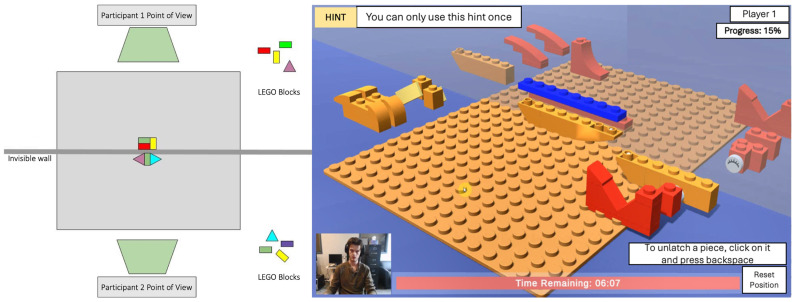
Layout of the environment (**left**). Point of view for Participant 1 (**right**).

LEGO-based tasks were chosen for the assessments because of their effectiveness in fostering collaborative skills, as demonstrated in previous studies on LEGO therapy and its adaptations [32,33,34]. The feasibility of these tasks was confirmed in a prior study [23]. Before the main assessment activity, participants would complete a tutorial task first. Participants first completed a simple virtual LEGO task individually to familiarize themselves with the user interface and game controls, following written and verbal instructions. Afterward, they would collaborate and work together in a synchronized virtual environment for the main assessment activity, where they viewed two object design variations (Figure 2) and had three minutes to discuss and agree on one to construct. The workspace allowed participants to see each other through video streaming, with LEGO pieces split equally and separated by a translucent wall (Figure 3). Each participant could only move pieces on their side and had to request specific pieces from their partner, promoting collaboration. Before they began the assembly, they were shown an image of the completed object for one minute for them to remember and share assembly information, then had 8 min to assemble it from memory, with the option to request a hint by viewing the completed object image again. No additional feedback was provided by the system.

### 2.4. Feedback Mechanism in the Training Task

Feedback mechanism is considered instrumental in skills development [35,36,37]. Commonly, feedback is delivered verbally by observers or therapists [38,39]. Although this allows for improved corrections, it is a laborious process that is prone to bias and inconsistencies [40,41]. Given that individualized feedback plays an important role in skill development to improve learning experiences in cross-neurotype interactions [42], researchers have explored automated or embedded feedback mechanisms [43,44,45], leveraging machine learning or AI-driven adaptive feedback mechanisms that adjust the feedback based on single-user performance. However, their role in collaborative interactions remains limited.

The feedback mechanism in VIRTUES provides individualized prompts to both participants when interaction breakdowns are detected, based on task performance and collaborative actions. In this work, real-time feedback refers to feedback delivered during task execution rather than after completion. The system continuously monitors multimodal data (e.g., speech, gaze, and task actions) to assess participant states. Feedback is not triggered immediately; instead, a 30 s wait time is applied to avoid disrupting natural collaboration. This delay allows participants time to resolve challenges independently and was determined in consultation with a behavioral analyst, balancing support with minimal interruption [46]. When triggered, the system delivers neutral, non-directive prompts via audio and on-screen messages (e.g., “Try checking in with your partner”). Prompts are adapted to each participant’s behavior to encourage reciprocal engagement. For example, a neurotypical participant may be prompted to initiate communication, while an autistic participant may be encouraged to check in with their partner. This mechanism serves as a collaborative scaffold, promoting balanced interaction and mutual understanding in cross-neurotype teams. The following paragraph elaborates on the design process.

**Figure 4:**
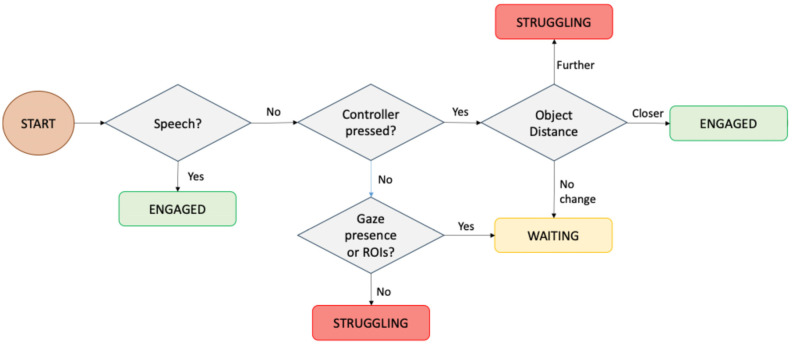
The participants’ behavior rule-based flow chart.

First, to support real-time feedback in VIRTUES, we defined a set of collaborative behaviors that could reflect varying levels of task engagement and interaction quality. A review of collaborative learning literature and consultations with behavioral analysts and stakeholders identified three core behavioral states most relevant for teamwork: *Engaged*, *Struggling*, and *Waiting* [22]. These were selected for their prevalence in collaborative tasks [47] and their potential to signal either positive interaction (*Engaged*) or moments needing support (*Struggling*, *Waiting*). Table 4 defines these behaviors in consultation with a certified behavioral analyst to guide consistent manual annotation and system-level classification. To automate the recognition of these behaviors in real time, we analyzed video data from a previous study [22] with a certified behavioral analyst to create a rule-based model that mirrors human annotation logic for classifying participants’ behaviors (Figure 4). The rule-based model classifies collaborative behaviors using features derived from speech, controller activity, object movement, and eye gaze. If speech was detected, the participant was labeled as *Engaged*. If there was no speech, the system checked for controller input; if present, it evaluated object movement: movement away from the target indicated *Struggling*, while movement toward the target indicated *Engaged*. If the object was stationary despite controller input, the behavior was labeled as *Waiting*. When both speech and controller input were absent, the model checked for eye gaze; the presence of gaze suggested *Waiting*, whereas absence indicated *Struggling*. This sequential logic enables simple, interpretable behavior classification in real time.

**Figure 5:**
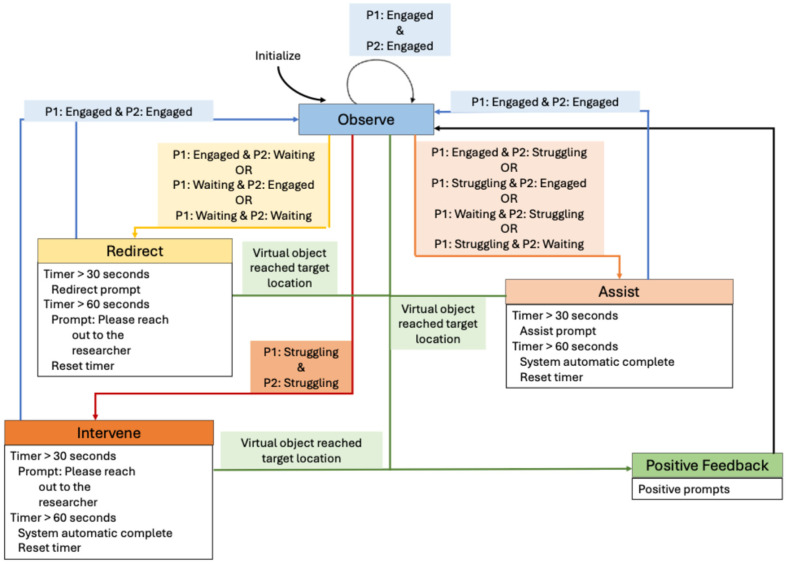
State transition diagram for feedback mechanism based on participants’ behavior.

Next, the feedback mechanism was modeled as a finite state machine (FSM) as shown in Figure 5. We chose FSM as it offers a structured model of social behavior rules, modularity of the state’s design, and enables real-time feedback based on real-time input [48,49,50]. The FSM was a quadruple deterministic model, with five states: Observe, Redirect, Assist, Intervene, and Positive Feedback adapting dynamically based on participants’ behaviors. Table 5 summarizes the FSM states.

The 30 s waiting window in the FSM was chosen based on guidance from behavioral analysts and previous work on the feedback mechanism [46]. This duration balances between autonomy and support, allowing participants enough time to process the situation and collaborate independently, without risking disengagement or frustration from excessive delays [51].

### 2.5. Experiment Design

The study involved 24 adult participants, comprising 12 autistic and 12 neurotypical (NT) individuals, who were paired into 12 ASD-NT dyads to reflect real-world workplace interactions. Autistic participants were recruited from Vanderbilt University’s Kennedy Center autism registry. Autistic participants and NT participants were recruited via word of mouth and flyers. Dyads were matched by gender to control for bias, resulting in 11 male pairs and 1 female pair, aligning with autism’s gender diagnosis disparity [52]. Six dyads were assigned to the training group and six to the control group.

The current study was designed as a single-session feasibility trial with a duration of approximately one hour per dyad. This condensed timeframe was selected to evaluate the immediate usability of the VIRTUES platform and the participants’ real-time responsiveness to multimodal feedback. Consequently, the analysis focuses on observing an immediate shift in collaborative behavioral profile and platform efficacy rather than longitudinal collaborative skill development.

**Figure 6:**
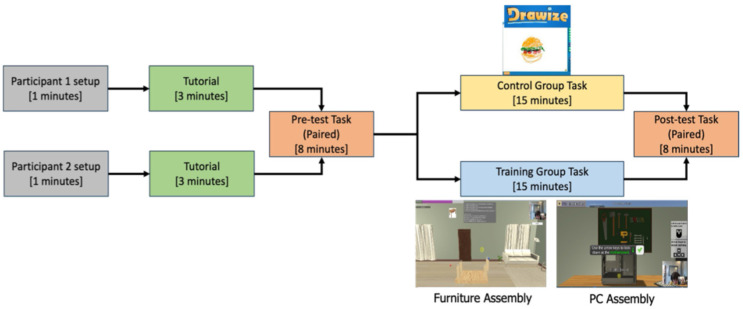
Experimental paradigm for the study.

The study followed a structured protocol (Figure 6). After providing informed consent, participants completed a tutorial to familiarize themselves with the virtual environment and controls. They then performed a pre-assessment task to establish baseline collaboration performance. The training group completed two virtual tasks (PC Assembly and Furniture Assembly) with a real-time feedback mechanism. The control group played Drawize [53], a web-based Pictionary game with no real-time feedback or audio-visual communication. This design choice was primarily guided by the need to establish a true baseline of naturalistic collaborative behavior in cross-neurotype dyads, unaffected by the novel sensory environment of the VIRTUES platform. Introducing the VR task to the control group without the feedback mechanism would have introduced a practice effect confound, where improvements in the post-assessment could be attributed to task familiarity rather than the absence or presence of feedback. By maintaining a treatment-free control, we isolated the holistic impact of the VIRTUES intervention (environment + real-time feedback) against standard interaction. Following the training, all participants completed a post-assessment task, and their collaboration performance was analyzed.

## 3. Methods

This subsection describes the steps that were taken to process the multimodal sensing data. This study adopts a dimension-based framework of collaboration to systematically analyze interaction patterns within VIRTUES. Multimodal sensing features were mapped onto these dimensions (Table 2) to enable a structured interpretation of collaborative behavior.

### 3.1. Data Processing and Handling

All multimodal data, including speech, gaze, and task-related logs, were preprocessed prior to analysis. The logfile was synchronized using timestamps to ensure alignment across modalities. Basic cleaning steps included removing invalid or corrupted entries and filtering out non-task-related segments.

Missing data were handled using a conservative approach. Instances with incomplete or missing values for key variables (e.g., task score, active participation) were excluded from the corresponding analysis. For intermittent missing values within a session (e.g., brief signal loss in gaze tracking), no imputation was performed; instead, analyses were conducted using available data to avoid introducing bias. Given the exploratory nature of the study and limited sample size, this approach prioritized data integrity and transparency.

### 3.2. Speech Data Analysis

Although speech data accounted for only 10% to 15% of the overall interaction, the extracted utterances provided valuable insights for assessing the communication aspects of teamwork and executive functioning. Real-time speech from both ASD and NT participants is transcribed using Microsoft Azure’s Speech-to-Text service, integrated into Unity via a continuous listener function [25]. Azure was selected for its high performance and low word error rate, particularly among individuals with accents or disabilities, as validated by existing literature [54,55,56]. Below, we describe two key features used in the analysis. The API segments words into a group of utterances based on silence detection or a 15 s threshold [25].

(1) *Number of initiations*: Two researchers developed a coding scheme to label speech transcripts, categorizing utterances as initiations if they: (1) introduced a new topic or (2) said something after 30 s of silence. We used 30 s to allow participants to process and respond either verbally or nonverbally to their partners, before treating it as a new initiation. Researchers labeled the log files individually, resolving discrepancies through discussion. They achieved 96% agreement, with the remaining 4% reconciled collaboratively.
(2) *Dialogue acts classification*: In a previous feasibility study [22], an annotation scheme adapted from a verbal behavior coding [57] was designed to classify the transcribed speech into one of the eight dialogue acts (DA) as listed in Table 6. For example, sentences like “it wants you to put that into the CPU” will be objectively categorized as “Inform” based on their linguistic structure. The reliability of this approach was confirmed by two human annotators, who achieved an initial inter-annotator agreement of 95% before reconciling all differences to reach a final agreement of 100%. Then, the labeled data was used to train a Bidirectional Encoder Representations from Transformer (BERT) model [58]. Noisy data labeled as “Out” was excluded from our analysis.

### 3.3. Gaze and Other Non-Verbal Data Analysis

The study utilized the Tobii Unity Eye Tracking SDK [26] to (i) continuously capture gaze points, and (ii) gaze fixation of approximately 200 ms [59] detected on pre-defined regions of interest (ROIs) or virtual objects. TobiiEyeX was selected based on its validated accuracy, latency, and sampling frequency, which are deemed suitable for evaluating active fixation within 3D virtual environments [60]. Before beginning the experiment, we calibrated the participants’ gaze using the Tobii Eye Tracking for Windows application [26].

After the experiment, the gaze data were sorted into three categories: (1) *Task-related gaze*, where the participants’ gaze focused on virtual objects or regions of interest; (2) *Social gaze*, where participants looked at the video streaming window within the virtual environment; and (3) *Time-related gaze*, where participants monitored the timer bar.

On the other hand, key parameters (as listed in Table 1) from the input controller were also used in our evaluation of VIRTUES. These parameters include: *Total Score*, *Individual Score*, *Piece Shared*, and *Active Effort*.

### 3.4. Multimodal Sensing Features Mapping to Dimensions of Collaboration

Quantitative and scalable assessment is crucial to accurately measure collaborative interactions. Objective assessment complements traditional assessments with data-driven insights that could minimize bias and errors [61,62,63]. Additionally, some of these quantitative measures are impossible for humans to measure through observation but can be easily acquired from sensors and system calculations, such as gaze duration and frequency, heart rate variability, the grip strength during manipulation, and the frequency of button presses. Echeverria et al. [64] developed a collaboration matrix linking multimodal measures to teamwork dynamics in a healthcare-based SBT and found that multimodal analytics can enhance group interaction assessments by providing objective insights that complement human observations. Recent work also emphasized the need for embedding human values in virtual mixed-reality design to ensure ethically grounded, inclusive experiences, especially in collaborative and therapeutic contexts [65].

To establish a direct link between multimodal sensing features that we analyzed and the defined dimensions of collaboration (Table 2), we developed a structured feature-to-dimension mapping framework. These features include the parameters that we captured in VIRTUES (Table 1) and the results of the analysis from 3.1 and 3.2.

Table 7 summarizes the mapping between multimodal sensing features and dialogue acts to be interpreted within a unified dimension of the collaboration framework.

### 3.5. Statistical Analysis

Statistical analyses were performed to evaluate the efficacy of VIRTUES across training and control groups, and to gain an understanding of collaboration behavior through quantitative multimodal sensing features. To address the nested nature of the dyadic data and the use of multimodal sensing devices, we applied an extensive statistical framework to the data. Normality was assessed using the Shapiro–Wilk test (*p* > 0.05) [66].

Linear mixed-effects regression models (LMM) [67] were used to account for the hierarchical structure of participants within dyads and the repeated measures from sensors in the pre- and post-task. Next, MANOVA [68] was used to assess the combined effects of pre- and post-tasks and control and training groups across multimodal sensing features, accounting for their interdependence. Then, a standard multiple linear regression [69] was used to identify behavioral predictors of task performance, focusing on the relationship between multimodal features and performance outcomes. Finally, paired and independent *t*-tests were conducted as supplementary analyses to provide descriptive comparisons and to facilitate interpretation of effect sizes.

For each outcome, we calculated the *p*-values, 95% Confidence Intervals (CI) for the mean difference, and Cohen’s d effect sizes to quantify the magnitude of training gains. Cohen’s d was interpreted as small (0.2), medium (0.5), and large (0.8). Furthermore, to control for the Type I error rate due to multiple comparisons across various sensing data, an adjusted *p*-value calculation was done using the Benjamini–Hochberg procedure [70]. As the study size was quite small and a single-session visit, a post hoc power analysis was also conducted using the *Task Score* as the primary feature

## 4. Results

### 4.1. Overview

The results present the statistical evaluation of the collaborative performance in VIRTUES, specifically the immediate efficacy of the training paradigm. Additionally, we are also looking at the underlying meaning of the multimodal sensing features within the predefined collaboration dimensions: (1) Dialogue Management, (2) Information Pooling, (3) Reciprocal Interaction, (4) Task Division and Coordination, and (5) Time Management.

### 4.2. Overall Efficacy of VIRTUES

The impact of the VIRTUES training paradigm on the collaborative performance was analyzed with a linear mixed-effects model. This analysis examines the effects of groups (Training vs. Control) and time (pre vs. post) on task performance, where dyad (ASD—NT) was included as a random effect to account for interdependence between paired participants. Table 8 summarizes this mixed-effect model. The analysis revealed a significant main effect of Time between pre- and post-assessment tasks (β = 14.17, *p*-value = 0.016, CI = [2.69, 25.64]), indicating that participants demonstrated improved task scores from pre- to post-assessment tasks. Another significant main effect was also observed for Neurotype (β = −13.75, *p*-value = 0.001, CI = [−21.86, −5.64]), which means that ASD participants’ scores vary significantly from NT participants. However, neither group’s effect (β = −14.17, *p*-value = 0.153, CI = [−33.61, 5.28]) nor group’s and time effect (β = 14.17, *p*-value = 0.087, CI = [−2.06, 30.39]) was statistically significant. This suggests that, although participants improved over time, the training group did not lead to significantly greater improvements compared to the control group.

**Figure 7:**
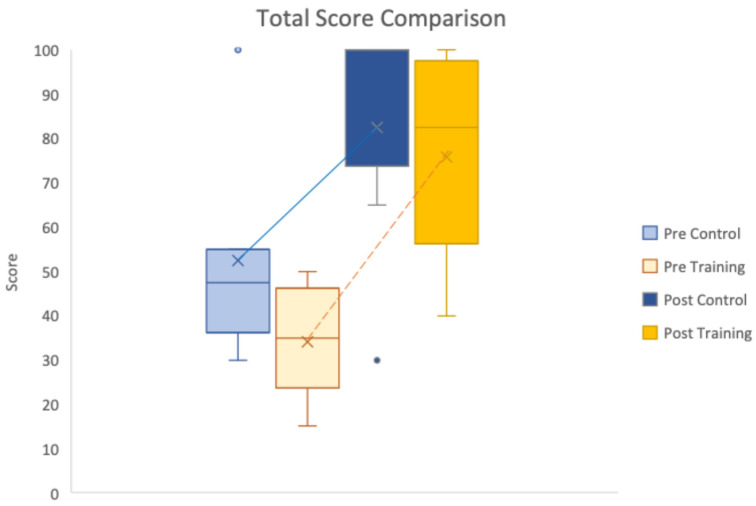
Total Score comparison between Control and Training groups across pre- and post-assessment tasks.

Table 9 consolidates key sensing features and the task score mean and standard deviation. The result revealed that while the Task Score in the pre-assessment task for the Training group was lower (M = 59.17, SD = 23.92) compared to the Control group (M = 73.33, SD = 20.60), the Training group achieved performance parity in the post-assessment task (M = 87.50, SD = 17.12). Figure 7 illustrates these findings through the larger Task Score improvement for the Training group from the steeper trajectory compared to the trajectory for the Control group.

*t*-test results compared linear changes between participants across time, neurotype, groups, and group and time. Table 10 supports the LMM result in Table 8 and Table 9. Based on the results, ASD participants (*p*-value = 0.014, d = 1.72) in the training group showed significant improvements in the post-test task, and while the NT participants in the training group did not show any significant changes, the effect is still quite large (*p*-value = 0.084, d = 1.56).

A post hoc power analysis was conducted for the *Task Score* to evaluate the adequacy of the dyadic sample size (*n* = 12 dyads). For the within-group improvement in the Training group, the analysis revealed an extremely large effect size (Cohen’s d = 1.92) and an observed power of 0.93, exceeding the recommended 0.80 threshold. For the comparison of gain scores between the Training and Control groups, a large effect size was observed (Cohen’s d = 1.00); however, the statistical power was 0.35, reflecting the pilot nature of this feasibility study. These results suggest that while the intervention’s impact is robust, future large-scale trials are needed to enhance the generalizability of the training paradigm.

### 4.3. Multimodal Predictors of Collaborative Dimensions

**Figure 8:**
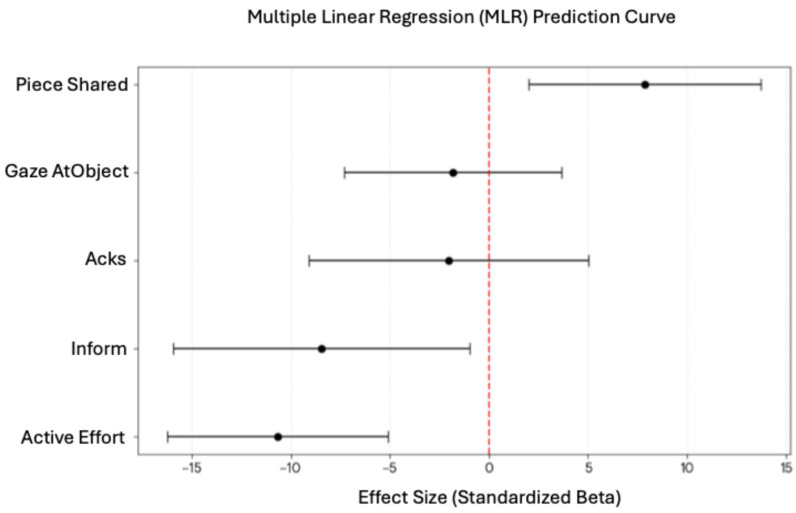
Multiple linear regression curve for key features as predictor of collaboration success.

Next, we wanted to further examine the relationship between multimodal sensing features and collaborative performance success. A multiple linear regression (MLR) analysis was conducted to establish the relationship and identify key features that could predict task success. Table 11 lists the regression model result that was statistically significant (F(4,43) = 6.84, *p*-value < 0.001). The model showed that Information Pooling (measured through *Piece Shared*) was a significant positive predictor (β = 7.87, *p*-value = 0.009), indicating that increased piece sharing and information sharing were strongly associated with improved task performance. Conversely, Task Coordination (represented by *Active Effort*) was a significant negative predictor (β = −10.67, *p*-value < 0.001, d = −1.195), suggesting that higher levels of effort may reflect task difficulty or inefficient interaction rather than effective collaboration. A similar pattern was observed for the dialogue act *Inform* (β = −8.46, *p*-value = 0.028, d = −0.704), indicating that as dyads become more synchronized with the task, the need for verbal instructions reduced. Gaze and speech Initiation was not a predictor of collaboration success. Figure 8 provides the visualized representation of this model.

### 4.4. Multivariate Effects on Collaborative Dimensions

**Figure 9:**
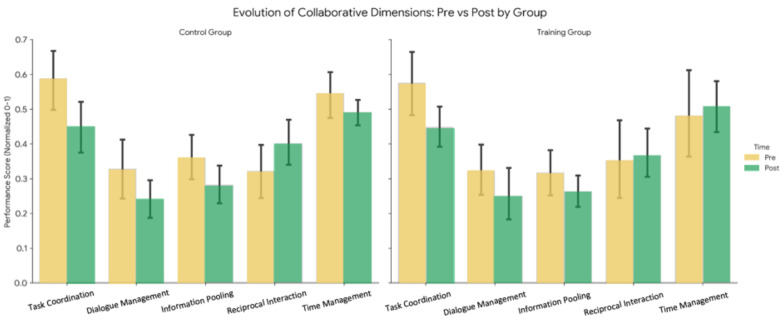
Side-by-side comparison of dimensions of collaboration between groups and across time.

A multivariate analysis of variance (MANOVA) was conducted to compare differences in collaborative performance across time, group, and neurotype. Table 12 consolidates the results of this analysis. A significant multivariate effect of time (Λ = 0.672, F(5,39) = 3.80, *p* = 0.007) was observed, indicating that the collaborative performance of all dyads changed significantly between the pre-assessment and post-assessment sessions. No significant multivariate effect of neurotype was observed, indicating that when considering all collaborative dimensions simultaneously, the behavioral profiles of ASD and Neurotypical (NT) participants were not fundamentally distinct in their multivariate structure. The same goes for the multivariate effect of group, suggesting that the training intervention did not produce differential effects across conditions. To visualize the dimensions of collaboration across groups and time, features were aggregated into the five dimensions of collaboration. The mean effect sizes were calculated by averaging the absolute Cohen’s d of constituent features. Figure 9 illustrates a side-by-side comparison of the collaborative dimensions between groups across pre- and post-assessment tasks. The pattern of each dimension of collaboration between the Training and Control groups is very similar to each other, supporting the results that no significant difference exists between the Training and Control groups.

### 4.5. Dialogue Acts Classification Based on Neurotype, Group, and Time

**Figure 10:**
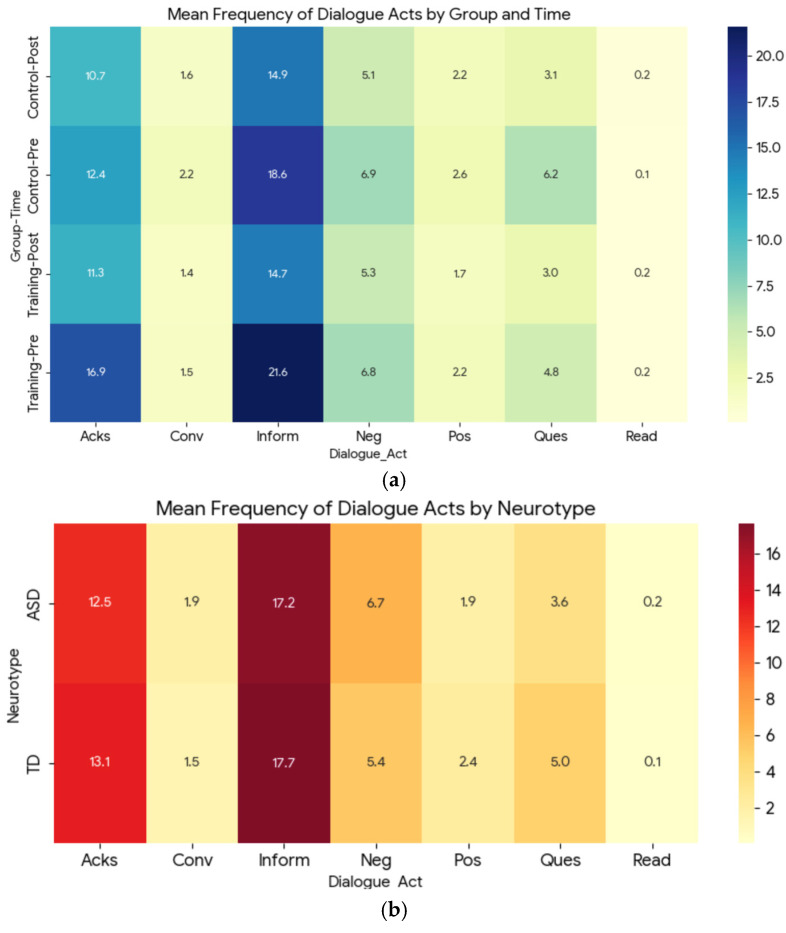
Dialogue act frequency heatmap for (**a**) group and time comparison and (**b**) neurotype comparison.

To provide us with a deeper understanding of how dialogue act patterns changed across both groups and pre- and post-assessment tasks, we generated a heatmap of the mean frequency of dialogue acts for Group and Time as shown in Figure 10a. The heatmap compares how the communication profile from pre- to post-assessment in both training and control groups shifted. The most frequent dialogue act across both groups is *Inform*, representing the core of the collaborative task—sharing and exchanging information. After the post-assessment task in the Training group, we could see a shift in the *Ques*, *Acks*, and *Inform* dialogue acts as an indication of improved coordination. Additionally, a heatmap was also generated for the mean frequency of dialogue acts by neurotype (Figure 10b). This highlights the baseline and overall communication difference between ASD and NT participants. However, there were no statistically significant differences across neurotype.

### 4.6. Task-Related Gaze Data Analysis

**Figure 11:**
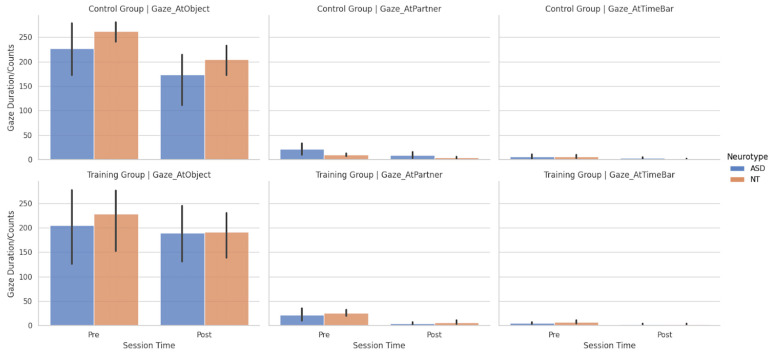
Gaze behavior shifts in participants in both Control and Training groups from pre- to post-assessment tasks.

Descriptive analysis of gaze behavior (Table 13) revealed a consistent downward trend in gaze counts across all targets from pre- to post-assessment tasks. Notably, Gaze at Partner showed a substantial reduction in the Training group for both ASD (M_Pre_ = 21.50 to M_Post_ = 4.33) and NT participants (M_Pre_ = 25.17 to M_Post_ = 6.17). This reduction was more pronounced in the Training group compared to the Control group (ASD: M_Pre_ = 21.50 to M_Post_ = 9.00). Similarly, Gaze at Object decreased across all cohorts, with the Control group showing a larger shift from a higher baseline (M_Pre_ = 262.00, SD = 28.73) to post-test levels (M_Post_ = 204.33, SD = 41.47). The shift is visualized in Figure 11.

## 5. Discussion 

This study investigated how multimodal sensing features map onto key dimensions of collaboration and how these dimensions relate to task performance in a collaborative virtual environment. The outcome of this study indicates that structured virtual collaboration, supported by targeted design elements, can foster balanced and reciprocal teamwork across neurotypes. By using the LMM, MLR and multivariate analysis, we were able to account for the inherent “dyadic chemistry” of each pair, providing a more rigorous validation of the VIRTUES platform.

Across our analyses, we consistently observe that participants demonstrated significant improvement over time, while no significant differences were observed between training and control groups. Prior studies have also shown the utility of virtual environments for capturing nuanced physiological and behavioral responses in autistic individuals, particularly in emotion recognition and engagement [71].

### 5.1. Multimodal Sensing to Represent Collaborative Dimensions

A core contribution of this work is the mapping of objective, multimodal sensing features to the defined dimensions of collaboration.

*Dialogue Management* was mapped to dialogue acts Acks, Ques, and the number of initiations. Although these features did not contribute any significant effects to the task completion, they form a stable interaction structure that supports collaboration that does not independently drive performance. For example, as dyads became more coordinated and familiar with the task, they required fewer verbal exchanges to achieve their goals. These findings could indicate that verbal communication serves a facilitative role rather than a causal role in task success.

*Information Pooling* was mapped to the Inform dialogue acts and *Piece Shared* feature as an indicator of information exchange happening. There was no significant effect coming from the Inform dialogue act; however, there was a significant effect associated with the *Piece Shared*. This suggests that information pooling is most effective when embedded within coordinated task actions, rather than reflected solely in communication frequency. This supports and extends the frameworks defined by Meier et al., showing that Information Pooling is most effective when tightly coupled with task execution [29].

*Reciprocal Interaction* was mapped to *Active Effort* and *Initiation Count*. *Active Effort* showed a significant increase over time (*p* < 0.001). However, regression analysis revealed that *Active Effort* was negatively associated with *Task Score* (β = −10.67, *p* < 0.001, CI = [−0.27, −0.08]). These findings suggest that increased participation does not necessarily translate into effective collaboration. Higher effort may reflect inefficiencies, confusion, or task difficulty, highlighting the distinction between engagement and productive contribution.

*Task Division* and *Coordination* emerged as the strongest determinant of task performance based on the *Piece Shared* effects. Mixed-effects modeling revealed significant improvement over time for *Piece Shared*, while regression analysis showed that *Piece Shared* was a strong positive predictor of performance. These findings indicate that effective collaboration is fundamentally grounded in the ability to coordinate task-relevant actions. This is expected since the collaborative tasks rely heavily on the coordinated movement of virtual objects.

*Time Management* was mapped to *Task Score*. In this study, there were significant effects of time from pre- to post- assessment task on the *Task Score*. We can interpret this as when dyads are able to achieve significant improvement in their *Task Score*, it means that they have good time management.

### 5.2. Impact of VIRTUES on Dyadic Collaboration Quality and Quantity

While participants in both groups improved in task scores, the LMM provides the key measure of performance. For example, from Table 8, *Piece Shared*, *Active Effort*, and *Inform* dialogue acts contribute the most to the collaborative performance. The significant improvement in the *Piece Shared* is a direct measure of collaborative action and was a strong positive predictor of performance. Meanwhile, *Inform* and *Active Effort* were negatively associated with performance, suggesting that increased effort may reflect inefficiency, confusion, or experiencing difficulty in performing the task. This finding highlights that task-related collaborative behaviors are critical in teamwork, rather than pure communication quantity. Traditional social skills training often focuses on increasing the quantity of social initiations. However, recent HCI-related research suggests that in collaborative tasks, implicit coordination, where participants rely less on explicit verbalization and more on shared environmental cues [72], is in line with our findings.

### 5.3. Reduced Gaze Counts as Indicator of Familiarity and Improved Efficiency

The gaze data showed a decrease in Gaze_atPartner and Gaze_atObject fixation counts. This could be indicative of increased task mastery, where participants would spend less time looking around and only focus on objects and areas that they are working on. In the early stages of collaboration (pre-test), participants across both neurotypes gaze more frequently to familiarize themselves with the virtual space and objects. However, by the post-test decline in gaze counts suggests that dyads had developed a shared mental model of the virtual space. This finding is consistent with the results presented for other task-related features, specifically for *Active Effort*. It indicates that VIRTUES enables participants to acclimate to the virtual environment and reduce the social cognitive load.

### 5.4. Feedback Mechanism Scaffolding Collaborative Performance

The present study did not include direct measures of feedback mechanism usage, such as prompt frequency, user responses, or qualitative feedback. Instead, an indirect assessment was conducted using performance outcomes. The consistent results we saw across various sensing features and statistical analyses suggest that the feedback mechanism scaffolded the transition from individual task-work to collective teamwork effort. Between-group comparisons revealed no significant performance decrement in the Training group (*p* > 0.05), indicating that the rule-based prompts did not negatively interfere with dyadic interaction or impose an overwhelming cognitive load. Furthermore, the Training group showed a significant improvement in collaboration scores from pre- to post-assessment tasks (*p* < 0.01, d = 1.92), an effect not observed in the control condition. These findings should be interpreted cautiously given the short study duration and limited sample size. Future work should incorporate detailed logging of feedback usage and qualitative user evaluations to better understand how participants engage with and perceive the prompts.

This alignment in task outcomes reflects a shift toward more equitable contributions and likely reflects mutual interactions taking place during interaction. These findings highlight the importance of the double empathy problem in understanding and addressing communication challenges between individuals from different neurotypes, emphasizing the need for fostering mutual understanding across neurotypes [73]. By providing neutral, non-directive prompts to both partners (e.g., “Try checking in with your partner”), VIRTUES encouraged mutual adaptation rather than placing the burden of “correction” solely on the autistic participant. These structured supports align with previous works to encourage collaboration and mutual adaptation [74,75].

### 5.5. Behavioral Pattern Analysis

Detailed analysis of the multimodal sensing features reveals a strong indication of coordination. From the dialogue acts analysis, *Inform* is the most frequent verbal exchange, which is part of Information Pooling and Task Coordination dimension. This verbal sharing was strongly coupled with physical action, as the *Piece Shared* metric emerged as the primary predictor of task success (*p* < 0.05, d = 0.836) and *Active Effort* is a negative predictor with a strong effect (*p* < 0.05, d = −1.195). Consolidated gaze data confirmed that participants maintained a consistent focus on shared virtual objects during these exchanges. Together, these markers define a pattern that is consistent with each other, where verbal, visual, and physical channels converge to represent implicit coordination to facilitate collaboration. Again, this supports the state-of-the-art research findings by Butchibabu et al. [72].

### 5.6. Feasibility and Limitations of VIRTUES as a Collaborative Training Tool

As the study was performed as a single-session feasibility and usability trial, the results of our analyses demonstrate the limited effect of the VIRTUES training paradigm and the feedback mechanism. Although we could not show any significant contribution of the training paradigm, based on the other findings, VIRTUES can reliably capture and support nuanced social-behavioral changes in a short window. The consistency across verbal (dialogue acts) and non-verbal (gaze, active effort, etc.) modalities points to the robustness of the multimodal architecture. Future work will explore longitudinal training with a larger sample size to determine if these dyadic efficiency gains, such as the reduced reliance on *Ques* and *Neg* dialogue acts, that represents Dialogue Management, Reciprocal Interaction, and Information Pooling, can be generalized to real-world workplace interactions beyond the VR environment.

While certain metrics, such as Task Score and Active Effort, showed significant improvement, other communicative behaviors (e.g., Initiation with Response, Gaze at Video) did not reach statistical significance. This is likely attributable to the limited exposure time; a single one-hour visit is insufficient for participants to fully internalize and automate new collaborative strategies. While the absence of negative effects suggests the platform is safe and well-tolerated, we cannot definitively claim that the feedback mechanism significantly ‘retrained’ the dyadic interaction within this timeframe. Future research should employ multi-session longitudinal designs to capture the latent period required for these behaviors to stabilize and show any significant improvements.

Some limitations that we should acknowledge from this study are listed below:

1. The sample size was small, with 12 ASD-NT dyads, which constrains the statistical power and generalizability of the results. A larger cohort is needed to validate these initial findings across broader populations. It would also benefit the study if we could introduce an ASD-ASD dyad and compare the results to the ASD-NT dyads, similar to how ref. [74] performed the telephone study.
2. The training duration was brief (20 min), which may limit the full potential impact of the intervention. Longer or repeated sessions could reveal more sustained behavioral changes.
3. Similarities between the pre- and post-assessment tasks may have caused a familiarity effect on the participant. Future iterations should consider counterbalancing or diversifying task types to reduce potential learning effects.
4. The feedback mechanism was based on a rule-based finite state machine, which lacks adaptability to nuanced, dynamic team interactions. A more flexible approach, such as a probabilistic or machine learning-based model, could improve the responsiveness and personalization of the feedback [76].

These limitations highlight areas for refinement in future studies to enhance the system’s reliability, scalability, and applicability. Nevertheless, these findings lay the groundwork for developing scalable, data-driven tools to support and assess neurodiverse teamwork in both research and applied settings.

### 5.7. Ethical Considerations

While this work demonstrates the potential of CVEs to enhance cross-neurotype collaboration, we carefully addressed key ethical considerations in the development of VIRTUES. The real-time feedback mechanism was designed to avoid reinforcing normative communication patterns that could marginalize neurodivergent traits. As such, we consulted two experienced behavioral analysts to create a supportive feedback mechanism protocol that is not biased.

To protect participant privacy, all behavioral data were securely stored, and informed consent was obtained from all participants. Importantly, neurodiverse individuals were also engaged in the design process to ensure VIRTUES remains inclusive and respectful across neurotypes.

## 6. Conclusions

Collaboration and teamwork are important skills that could improve productivity and group performance if done well. However, there is limited research that focuses on cross-neurotype collaboration—that comes with its own unique challenges. This study introduced VIRTUES, a CVE-based training system specifically aimed at supporting cross-neurotype collaboration. VIRTUES was designed to support and assess collaborative performance in cross-neurotype interactions. Through a combination of structured collaborative assessment tasks, multimodal sensing data, and a rule-based feedback mechanism, VIRTUES provides a novel, scalable virtual framework for promoting and assessing collaborative interactions.

In this work, we (1) defined five measurable dimensions of collaboration that are both verbal and non-verbal; (2) designed a set of training and assessment tasks based on these dimensions, (3) mapped these dimensions to measurable multimodal sensing data to enable quantifiable analysis of collaborative performance; (4) developed a rule-based feedback mechanism based on multimodal sensing data integrated into the virtual environment to support dyadic interaction in VIRTUES; and (5) conducted a controlled user study involving 12 ASD-NT dyads, evaluating the immediate effects of the feedback mechanism.

The results of this study demonstrate that the VIRTUES framework for supporting and quantifying collaborative fluency in cross-neurotype dyads is achievable, given that our sample size is small. By employing a multimodal sensing architecture, we moved beyond traditional outcome-based metrics to reveal the underlying behavioral mechanisms of teamwork. Our findings, particularly the significant interaction effect in resource sharing and the inverse relationship between physical effort and task success, suggest that effective collaboration in virtual environments is driven by a transition from high-effort verbal negotiation to coordinated sharing of objects. While this feasibility study is limited by its single-session design and sample size, the parity achieved by ASD-NT dyads suggests that structured CVEs can serve as a cross-neurotype social bridge. These results lay the groundwork for future longitudinal interventions aimed at fostering inclusive, high-efficiency teamwork in neurodiverse workplaces and educational settings.

Future work will include expanding the sample population and extending the training duration to multiple visits to investigate long-term learning outcomes. Another focus could be on expanding the objective measures of the dimensions of collaboration by combining quantitative sensing features with qualitative measures by observers or trainers. Finally, evaluating an adaptive feedback mechanism in a training paradigm could provide us with meaningful data on how best to support cross-neurotype collaboration.

## Figures and Tables

**Table 1:** Multimodal sensing data acquisition for measuring dimensions of collaborations.

No.	Parameter	Description	No.	Parameter	Description
1	Timestamp	Timestamp in HH:mm:seconds format	9	Piece at target	Name of piece placed on target
2	Player Label	Either Player 1 or Player 2	10	Piece Shared	Name of piece shared with the other player
3	Transcribed Text	Transcribed speech	11	Shared Count	Number of pieces shared by each player
4	X-gaze point	The x-coordinate of the gaze points	12	Piece Selected	Name of the object selected
5	Y-gaze point	The y-coordinate of the gaze points	13	Active Effort	A logic value indicating active (1) or inactive (0) interaction with the system
6	Focused Object	Name of the object the participant was looking at	14	Game Duration	Amount of time spent on the task
7	Total Score	The overall percentage of pieces on target by both participants	15	Object Distance	Distance of the object from the target location
8	Individual Score	Percentage of pieces on target by each participant			

**Table 2:** Updated dimensions of collaboration for VIRTUES.

Dimensions	Updated Definitions
Dialogue Management	Participants engage in back-and-forth communication and activities.
Information Pooling	Participants share information with each other. Participants either provide or ask for information. * *Sustaining mutual understanding is embedded within this dimension as showing understanding can happen while exchanging information.*
Reciprocal Interaction	Measure of participants’ contribution and how they are distributed. * *Individual task orientation is combined with this dimension as a contribution to the task, which is representative of their motivation.*
Task Division and Coordination	Participants discussed and assigned tasks between them. Participants’ ability to maneuver in the task. * *Reaching consensus and technical coordination are embedded within this dimension as decision making can happen while dividing the task*
Time Management	Participants display awareness of time constraints and planning.

* Italicized texts represent the original dimensions that were integrated into the main dimensions.

**Table 3:** Description of collaborative training tasks.

Tasks	Tasks Description	Embedded Dimensions of Collaborations
PC Assembly	Two participants work together to attach various virtual computer hardware to build a computer within the allocated time. Participants were given installation instructions and access to virtual hardware pieces. They used the keyboard and mouse to select and move the virtual hardware to the correct location.	**Dialogue management**—Some installation steps were printed in a different language, requiring participants to read out the English instructions to each other as they progress in the task. **Information pooling**—Each participant was given access to different sets of hardware and installation manuals which required them to exchange installation information. **Reciprocal interaction**—Participants would need to take turns to attach the different components they have in order as mentioned in the instructions. **Task division and coordination**—Participants were given different points of view that limited their view when trying to attach the hardware in place. They had to coordinate their movement to place the hardware correctly. **Time management**—Participants were allocated a limited time to complete the task and thus needed to plan and manage the task within the given time.
Furniture Assembly	Two participants needed to work together to assemble various pieces of furniture either by following a given set of written instructions or an image of the completed furniture. They used the haptic device, Touch [34], to manipulate the furniture parts.	**Dialogue management**—The participants would need to verbally read the instructions to each other when available or describe the look of the furniture when no instructions were available while putting the furniture together. **Information pooling**—The installation instructions for each participant were different and had missing key information only available to the other participant. **Reciprocal interaction**—Participants had to plan and strategize how they were going to assemble the furniture when no written instructions were given. **Task division and coordination**—One part of the instruction explicitly mentions which participant needs to move which part. For example, “Player 1 attach leg 3 of the table to the blue pad” and “Player 2 attach leg 1 of the table to the white pad on the table”. **Time management**—Participants had limited time to complete the task and needed to plan and divide the task to finish in time.

**Table 4:** Collaborative behaviors in dyadic interactions.

Behavior	Definition	Multimodal Sensing
Engaged	Participants are interacting with their partner and performing the task.	Measured from transcribed speech and controller input data
Struggling	Participants are unable to correctly place an object in the correct location. Participants are not responding to their partners. Participants are not focusing their gaze on any object or region of interest.	Measured from transcribed speech, task progression, gaze data, and controller input data
Waiting	Participants are not performing any task but still maintain their gaze on the region of interest.	Measured from task progression, gaze data, and controller input data

**Table 5:** Feedback mechanism, FSM definition and description.

States	Definition	Action
Observe	When both participants are actively *Engaged*, the system remains in this state	No action
Redirect	If one participant is *Waiting* (e.g., during turn-taking or after completing a task while their partner is still performing the task), the system transitions to this state	After 30 s, and the participant is still *Waiting*, VIRTUES issues two complementary prompts: To the *Waiting* participant: “Please wait for your partner to finish” To their partner: “Your partner is waiting. Please update them on your progress.” If the *Waiting* behavior persists for another 30 s, or both participants become idle, the system prompts both users to request help from the researcher.
Assist	If one participant is flagged as *Struggling*, the system transitions to this state	After 30 s: The *Struggling* participant receives: “Try asking your partner for advice.” The partner is prompted: “Your partner seems to be struggling, please assist them.” If the *Struggling* behavior persists for another 30 s, VIRTUES automatically completes the current step in the task.
Intervene	If both participants are in *Struggling* behavior, the system enters this state	Prompts both participants to request help from the researcher, and after an additional 30 s, the system automatically completes the step in the task (similar to Assist state).
Positive Feedback	Upon successful completion of a step in the task or the overall task, VIRTUES provides positive reinforcement to both participants	VIRTUES prompts participants with: “Good job!”, “Great job working together!” or “Well done on working together to complete the task!”), then returns to the Observe state.

**Table 6:** Dialogue acts classes used for classification.

Label	Definition	Example
Acks	Indicate agreement or acknowledge	‘I know’, ‘You’re right’, ‘Okay’, ‘Yeah’, ‘Yup’, ‘Cool’, ‘uh-huh’, etc.
Neg	Disagree, confused, negative statements	‘No, I don’t need this one’, ‘I don’t think this is the right one’, ‘No’, ‘Um, I’m not sure’, ‘I don’t think so’, ‘Oh no’
Pos	Positive feedback from one participant to another	‘Well done’, ‘Good job’
Ques	Questions	‘What do you see?’, ‘Can you try W?’
Read	Any indication that the participant is reading task instructions	’Mine says to select the 8 gigabyte RAM’
Inform	Describe action or intention. Action directive statements or statements of instruction from one participant to another. Personal statements of opinion or non-opinion.	‘Try moving it more to the right’, ‘And then backward’, ‘Let’s see’, ‘Mine has me moving’, ‘Let me try’ ‘I think’, ‘I feel’, ‘I believe’, ‘I mean’, etc.
Conv	Conventional pleasantries	‘Thanks’, ‘Thank you’, ‘Sorry’, ‘My bad’
Out	Uninterpretable. When utterance is incomplete or does not make sense to the coder	‘The.’, ‘It said an end then snow’

**Table 7:** Multimodal sensing feature mapping to dimensions of collaboration.

Dimensions	Multimodal Sensing Features	Rationale
Dialogue Management	Number of initiations Dialogue acts: Acks, Neg, Conv	The number of conversations initiated and dialogues within these labels indicate flow and effective back-and-forth communication
Information Pooling	Piece Shared Dialogue acts: Ques, Read, Inform	The number of objects that were shared is an indication of information exchange, while the dialogue acts solidify this further as they indicate information gathering and exchanging happening.
Reciprocal Interaction	Active Effort Dialogue acts: Pos, Acks	The active effort tracks whenever a participant is actively participating in the task—a high-level indication of task participation. These dialogue acts then show any reciprocity between the participants.
Task Division and Coordination	Region of interests (ROIs) on virtual objects, Piece Shared, Active Effort Dialogue acts: Inform	The gaze data tracks visual coordination of the task, while pieces shared and active effort represent coordinated action. The dialogue act Inform indicates verbal instruction or division of task.
Time Management	Regions of interest (ROIs) on time bar	Time awareness is only tracked from the gaze data in the current design.

**Table 8:** Mixed-effect model results for VIRTUES.

Outcome	Beta (β)	SE	z-Value	*p*-Value *	CI
Group (Control and Training)	−14.17	9.92	−1.43	0.153	[−33.61, 5.28]
Time (Pre and Post)	14.17	5.85	2.42	0.016	[2.69, 25.64]
Neurotype (ASD and NT)	−13.75	4.14	−3.32	0.001	[−21.86, −5.64]
Group and Time	14.17	8.28	1.71	0.087	[−2.06, 30.39]

* All *p*-values corrected using the Benjamini–Hochberg procedure.

**Table 9:** Task performance and multimodal sensing features mean and standard deviation across groups and time.

Feature	Control Pre	Control Post	Training Pre	Training Post
Task Score	73.33 (20.60)	87.50 (20.50)	59.17 (23.92)	87.50 (17.12)
Piece Shared	6.00 (2.83)	5.08 (2.50)	4.00 (2.13)	4.83 (1.27)
Active Effort	291.92 (53.93)	203.08 (37.52)	278.17 (74.66)	220.00 (38.24)
Gaze at Object	244.33 (56.24)	188.67 (58.87)	216.83 (96.74)	190.00 (68.22)
Inform (Speech)	18.58 (11.86)	14.92 (6.76)	21.58 (13.17)	14.67 (6.67)

**Table 10:** *t*-tests comparing within-group and between-group Task Score performance.

Comparison	t-Stat	*p*-Value	Cohen’s d
ASD Training: Pre vs. Post	3.71	0.014	1.72 (Large)
NT Training: Pre vs. Post	2.15	0.084	1.56 (Large)
ASD: Training vs. Control (Post)	−0.12	0.908	−0.08

**Table 11:** Linear regression model showing key features that drive task performance.

Predictor	Beta (Std)	t-Stat	*p*-Value	Cohen’s d
Piece Shared	7.87	2.71	0.010	0.836
Active Effort	−10.67	−3.87	<0.001	−1.195
Inform (Speech)	−8.46	−2.28	0.028	−0.704
Gaze at Object	−1.82	−0.67	0.507	−0.207

**Table 12:** MANOVA comparison across time, group, neurotype, and combination of group and time.

Effect	Wilks’ Lambda (Λ)	F-Value	df (Num, Den)	*p*-Value
Time	0.672	3.80	5, 39	0.007
Neurotype	0.797	1.98	5, 39	0.103
Group	0.978	0.18	5, 39	0.969
Group and Time	0.943	0.47	5, 39	0.795

**Table 13:** The average gaze counts across the three primary categories, segmented by study group, neurotype, and session time.

Group	Neurotype	Time	Gaze at Object (M ± SD)	Gaze at Partner (M ± SD)	Gaze at TimeBar (M ± SD)
Control	ASD	Pre	226.67 ± 73.37	21.50 ± 16.02	5.83 ± 5.95
		Post	173.00 ± 72.92	9.00 ± 8.79	2.33 ± 2.73
	NT	Pre	262.00 ± 28.73	9.50 ± 3.56	5.83 ± 4.12
		Post	204.33 ± 41.47	3.33 ± 2.73	1.33 ± 1.03
Training	ASD	Pre	205.17 ± 107.91	21.50 ± 17.69	5.33 ± 2.58
		Post	189.17 ± 81.88	4.33 ± 3.67	2.17 ± 2.04
	NT	Pre	228.50 ± 92.85	25.17 ± 9.30	7.17 ± 4.92
		Post	190.83 ± 59.44	6.17 ± 5.56	2.50 ± 2.43

## Data Availability

The data presented in this study are available on request from the corresponding author. The data are not publicly available due to the privacy of our participants and the requirements of our IRB.
